# Is a Brief Online Booklet Sufficient to Reduce Fear of Cancer Recurrence or Progression in Women With Ovarian Cancer?

**DOI:** 10.3389/fpsyg.2021.634136

**Published:** 2021-02-25

**Authors:** Poorva Pradhan, Louise Sharpe, Phyllis N. Butow, Allan Ben Smith, Hayley Russell

**Affiliations:** ^1^School of Psychology, Faculty of Science, The University of Sydney, Sydney, NSW, Australia; ^2^Centre for Oncology Education and Research Translation, Ingham Institute for Applied Medical Research, South Western Sydney Clinical School, University of New South Wales, Kensington, NSW, Australia; ^3^Ovarian Cancer Australia, Queen Victoria Women's Centre, Melbourne, VIC, Australia

**Keywords:** cancer, oncology, neoplasm, fear of cancer recurrence, fear of cancer progression, ovarian cancer, psychoeducation

## Abstract

**Background:** Fear of cancer recurrence or progression (FCR/P) is a common challenge experienced by people living with and beyond cancer and is frequently endorsed as the highest unmet psychosocial need amongst survivors. This has prompted many cancer organizations to develop self-help resources for survivors to better manage these fears through psychoeducation, but little is known about whether they help reduce FCR/P.

**Method:** We recruited 62 women with ovarian cancer. Women reported on their medical history and demographic characteristics and completed the Fear of Progression Questionnaire-Short Form (FoP-Q-SF). They then read a booklet on FCR specifically created for Ovarian Cancer Australia by two of the authors (ABS and PB). One week after reading the booklet, 50/62 women (81%) completed the FoP-Q-SF and answered questions about their satisfaction with the booklet.

**Results:** More than half of the women (35/62; 56.5%) scored in the clinical range for FCR/P at baseline. Of the completers, 93% said that they would recommend the booklet to other women. Satisfaction with the booklet was relatively high (75.3/100) and more than two-thirds of women rated it as moderately helpful or better. However, FCR/P did not change significantly over the week following reading the booklet [*t*_(49)_ = 1.71, *p* = 0.09]. There was also no difference in change in FCR/P between women in the clinical vs. non-clinical range on the FoP-Q. Women high in FCR/P rated the booklet as less helpful in managing FCR/P (*r* = −0.316, *p* = 0.03), but overall satisfaction with the booklet was not associated with degree of FCR/P (*r* = −0.24, *p* = 0.10).

**Conclusions:** These results suggest that a simple online FCR booklet is acceptable to women with ovarian cancer and they are satisfied with the booklet, but, it was insufficient to change in FCR/P levels. These results suggest that such resources are valued by women with ovarian cancer, but more potent interventions are necessary to reduce FCR in this population.

## Introduction

Ovarian cancer is the leading cause of death among gynecological cancers with a 46% 5-year survival rate, as the disease is often diagnosed at an advance stage (Australian Institute of Health and Welfare, 2020). Approximately 70% of women with ovarian cancer are expected to experience recurrence of their cancer, particularly when diagnosed at later stages (Ovarian Cancer Research Alliance, 2020). Not surprisingly given this high recurrence rate, fear of cancer recurrence or progression (FCR/P) is one of the most common psychosocial concerns reported by this population (Matulonis et al., 2008; Kyriacou et al., 2017). FCR/P, defined as “fear, worry, or concern about the cancer returning or progressing” (Lebel et al., 2016, p. 3267), continues to be the most cited unmet need for ovarian cancer survivors (Tan et al., 2020). In a systematic review of FCR/P in ovarian cancer, Ozga et al. (2015) confirmed that FCR/P was prevalent amongst ovarian cancer survivors, and that women with ovarian cancer felt that there was insufficient support for managing FCR/P. Moreover, in a large prospective study of heterogeneous cancer survivors, those with advanced disease or who had experienced a recurrence had higher levels of FCR (Savard and Ivers, 2013).

Studies have identified that higher levels of FCR/P are associated with reduced quality of life (Hart et al., 2008), increased anxiety and depressive symptoms (Humphris et al., 2003; Koch et al., 2014) as well as post-traumatic stress symptoms (Mehnert et al., 2009). In addition to psychological symptoms, FCR/P is also characterized by increased healthcare costs (Thewes et al., 2012) and frequent reassurance seeking, such as through additional oncology appointments and increased medication use (Lebel et al., 2013). Therefore, individuals experiencing high levels of FCR often require specialized psychological support and intervention (Butow et al., 2018).

Despite clear evidence that high FCR/P is associated with poorer psychological outcomes and additional medical costs, specific interventions to manage FCR/P are still relatively scarce. In a meta-analysis of RCTs, Tauber et al. (2019) found over 23 controlled trials that had examined the efficacy of a psychological intervention and measured FCR, however, only 8 of these had specifically targeted FCR/P. The majority of those evaluated face-to-face interventions (e.g., ConquerFear, Butow et al., 2018) or blended interventions where treatments were administered partially online and partially face-to-face (e.g., SWORD, van de Wal et al., 2017). Both of these interventions required highly trained therapists and considerable time commitment (minimum of four sessions). In that meta-analysis, there were only two trials of a self-administered approach (i.e. minimal intervention). The study by Otto et al. (2016) found that such self-guided gratitude training interventions promoted well-being leading to a decrease in death-related FCR. The other intervention used Cognitive Bias Modification (CBM), an approach than aims to change implicit cognitive processes, such as interpreting ambiguous situations in a threatening way and preferentially attending to threatening information. The CBM approach was associated with reductions in health-related worries compared to placebo (Lichtenthal et al., 2017). One other randomized controlled trial, by Dieng et al. (2016), with melanoma survivors combined psychoeducational materials, as well as three telephone consultations with a psychologist, and found improvements in FCR/P, which were maintained at 12 month follow-up (Dieng et al., 2019). However, the telephone support still required specialist psycho-oncology skills. Given the number of survivors, and the fact that help with FCR/P remains a leading unmet psychosocial need, most services do not have the capacity to support all survivors with elevated levels of FCR/P.

Consequently, researchers are investigating other ways to increase access to information that might reduce or prevent persistent FCR/P. For example, brief interventions led by health professionals who manage the medical needs of survivors (most commonly nurses) have been developed. A recent systematic review of these approaches found that evidence to support their use is still lacking (Liu et al., 2019). Similarly, there has been interest in developing internet-delivered interventions specifically targeting FCR. Most of these are either in early stages of development (Smith et al., 2020) or currently being tested (e.g., Lyhne et al., 2020) and the only online intervention which specifically targeted FCR/P produced largely null results (van Helmondt et al., 2020).

Self-help materials have been used for other survivorship issues, including to reduce anxiety and depression and/or to improve quality of life. Cuthbert et al. (2019) identified 41 studies of self-help interventions that had been evaluated in randomized controlled trials. The results were largely mixed, with some showing short-term benefits and others showing little improvement in outcomes. None of these studies targeted FCR/P.

However, even in the absence of evidence, several non-profit organizations such as, Cancer Council Australia, National Breast Cancer Foundation, Breast Cancer Network Australia and Lymphoma Australia have developed online booklets or leaflets for addressing concerns related to cancer coming back or progressing. Whether these self-help materials attenuate FCR/P has not been the subject of research. Lynch et al. (2020) have recently completed a preliminary evaluation of a stepped care approach for survivors of melanoma who were treated with novel immunotherapies. The first step in their “FearLESS” program was a self-help intervention. Of those who scored in the sub-clinical range and were offered self-help, 90% did not feel the need for referral to individual therapy at the end of the study (Lynch et al., 2020). However, the authors did not evaluate whether changes in FCR/P were significant for those who received the self-management approach.

The evidence examining informational needs of cancer survivors suggests that most patients want to receive as much information as possible about their disease and its consequences (Shea–Budgell et al., 2014; Fletcher et al., 2017). A systematic review of 10 studies that assessed a range of patient outcomes in RCTs of educational resources specific to cancer, found that the provision of psychoeducation was associated with better outcomes for satisfaction, symptom management and anxiety and depressive symptoms (McPherson et al., 2001). However, we could not identify a purely psychoeducational resource that had been developed specifically for FCR/P which had been evaluated in terms of its acceptability and effect on FCR/P.

Therefore, we (PB & ABS) developed a simple online booklet that (a) outlined the nature of FCR/P, (b) provided information about how FCR/P becomes persistent, (c) suggested strategies (based on evidence-based treatments) that might help survivors to better manage FCR/P; and (d) provided links to where survivors can find additional help. The aims of this study were to determine whether (i) the booklet was acceptable to survivors (ii) survivors were satisfied with the booklet and would recommend it to others; and (iii) the booklet reduced levels of FCR/P.

It was hypothesized that

- Women with ovarian cancer will be satisfied with the booklet and would recommend it to other survivors.
- Women with ovarian cancer will have lower levels of FCR/P a week after reading the booklet compared to baseline.
- The booklet will lead to a greater reduction in FCR/P for women with low to mild FCR/P.

## Method

### Design

Women with ovarian cancer completed measures of FCR before and 1 week after reading an online psychoeducational booklet about FCR/P. In addition, a measure of satisfaction was given 1 week after women accessed the booklet.

### Participants

Women who had been diagnosed with ovarian cancer, were over 18 years of age, and fluent in English were eligible to take part in the study. Participants were recruited online through Ovarian Cancer Australia (OCA) (see below). Ethical approval was provided by the University of Sydney's Human Research Ethics Committee (Project no.: 2018/993). Informed consent was obtained from all participants online, and they were free to withdraw from the study at any time.

### Procedure

The new online FCR booklet developed by the authors was released through OCA and advertised to its members. When women indicated they would like to access the booklet, a pop-up window asked whether they would like the option of taking part in some research to evaluate the impact of the booklet on FCR/P. Women who chose not to do so, were directed immediately to the booklet, while those who indicated their interest in taking part in the research were invited to follow a link which described the study in more detail. Unfortunately, we were unable to get information from women who chose not to take part. After providing consent, participants were directed to an online questionnaire including some demographic and medical information and a measure of FCR/P^1^. On completion, women were given access to the booklet. One week later participating women were sent an email and asked to complete measures of FCR/P and satisfaction with the FCR/P booklet. We chose 1 week as a time frame because we suspected that any impact on FCR/P would be short-term, consistent with the systematic review on psychoeducational approaches (Cuthbert et al., 2019).

### Fear of Cancer Recurrence Booklet

The booklet was developed in conjunction with OCA and input from oncology health writer in terms of translating information from ConquerFear study suitable for women with ovarian cancer. It aims to provide information on FCR/P, which is identified as a significant survivorship issue for women with ovarian cancer (Kyriacou et al., 2017), and also suggest strategies to manage these fears. The techniques to manage FCR in this booklet were adapted from the ConquerFear program by Butow et al. (2017). See Table 1 for the list of contents in the booklet (online link to the booklet: https://www.ovariancancer.net.au/page/94/support-resources).

**Table 1:** List of contents in Fear of Recurrence booklet.

1. What does “cancer recurrence” mean?
2. Why are women fearful?
3. Types of fears
4. Common worry times
5. Day-to-day approaches to managing your fears
6. Carers' feelings
7. Some techniques for managing the fear of recurrence
8. Finding information online
9. Further information and support

### Materials

#### Satisfaction Questionnaire

The satisfaction questionnaire has three items that assess: satisfaction with the information provided in the booklet; helpfulness for managing the concerns about cancer coming back or progressing; and whether women would recommend it to another woman diagnosed with ovarian cancer. The participants rated each item on a 10-point scale, from 1 (not at all) to 10 (completely). A higher score indicates that women are more satisfied with the booklet. Women completed this questionnaire 1 week after reading the booklet.

#### Fear of Cancer Recurrence/Progression

The 12-item Fear of Progression Questionnaire- Short Form (FoP-Q-SF; Herschbach et al., 2005) was administered to assess the level of FCR/P. Responses options ask how often a particular symptom of FCR/P is experienced on a five-point scale from 1 (never) to 5 (very often) (5). Thewes et al. (2012) conducted a systematic review of assessment measures for FCR/P and recommended the use of the Fear of Cancer Recurrence Inventory (Simard and Savard, 2009) and the FoP-Q-SF for assessing FCR/P. We opted to use the FoP-Q-SF because for women with ovarian cancer, many of whom have already experienced a recurrence, fear of recurrence is less relevant than fear of progression. Scores on FoP-Q-SF range from 12 to 60 and a score of 34 and above is taken to indicate a clinical level of FoP (Herschbach et al., 2010). The Cronbach's alpha for the current sample was 0.85.

### Data Analysis

All statistical analyses were conducted in SPSS version 26. Preliminary analyses compared those women that completed the study vs. those who accessed the booklet but did not complete questionnaires after reading the booklet. For continuous variables, we used independent *t*-tests and for other variables we used Mann Whitney U tests (categorical variables) or Chi-square (dichotomous).

Mean scores and frequencies were examined for satisfaction ratings. For FCR/P, a paired samples *t*-test was used to compare the level of FCR/P before and after reading the booklet. Using the cut-off of 34 on the FoP-Q, we identified women with clinically significant levels of FCR/P vs. those who scored in the normal range to determine whether clinical FCR/P affected the impact of the booklet. To investigate the impact of clinical status, we conducted a mixed-model 2 (FCR/P: Clinical range vs. within normal range) x 2 (time: before vs. after reading the pamphlet) ANOVA. Finally, we conducted correlations between FCR/P and satisfaction ratings to determine whether level of FCR/P affected the satisfaction that women reported after reading the booklet.

## Results

### Participant Characteristics

Sixty-two women diagnosed with ovarian cancer were recruited for the study. Participants had a mean age of 56.9 years. In terms of stage of disease, relatively few women had Stage I (*n* = 10; 16%), or Stage II (*n* = 11; 18%) disease, with 47% (*n* = 30) reporting Stage III and 9 (15%) reporting stage IV cancer. See Table 2 for demographic and medical details. Of the 62 participants who commenced the study, 50 (19% attrition rate) completed the questionnaires again a week after reading the pamphlet.

**Table 2:** Demographic and clinical characteristics of the sample.

	**Cancer patients (*n* = 62)**
**Variable**	**Mean**
Age	56.9 (11.64)
Time since diagnosis	3.45 (3.29)
	**Frequency (percentage)**
Marital status	
Married	41 (65.45%)
Widowed	2 (3.64)
Divorced	9 (14.55)
Separated	3 (5.45)
Never married	7 (10.71)
Children	
None	13 (20.97)
One	9 (14.52)
Two	32 (51.61)
More than two	8 (12.9)
Education level	
Did not complete high school	0 (0)
Completed high school	24 (38.18)
Undergraduate degree at university	22 (36.36)
Postgraduate degree at university	16 (25.45)
Employment status	
Currently employed	28 (45.16)
Currently unemployed	34 (54.83)
Stage at diagnosis	
Stage 1	10 (16.36)
Stage 2	11 (18.18)
Stage 3	30 (47.27)
Stage 4	9 (14.53)
Not known	2 (3.64)
Current cancer status	
Currently on treatment	18 (29.09)
Active disease	2 (3.64)
In remission	42 (67.27)
Cancer recurrence	
Yes	22 (36.36)
No	40 (63.64)
Surgery	
Yes	1 (1.12)
No	61 (98.88)
Treatment type	
Radiotherapy	0 (0)
Chemotherapy	46 (74.19)
Hormonal therapy	12 (19.35)
No treatment	4 (6.45)
CA-125 testing	
Yes	60 (96.23)
No	2 (3.77)
Not known	0 (0)

Between group comparisons revealed that there was no significant difference between participants who completed the study and those who did not for age [*t*_(60)_= 1.13, *p* = 0.26], education (*U* = 216, *p* = 0.11), cancer stage (*U* = 276, *p* = 0.65), number of children (*U* = 289.5, *p* = 0.84), marital status (*U* = 284, *p* = 0.73), cancer status [$\chi_{\left(1,62\right)}^{2}=$ 1.06, *p* = 0.33] or employment status [$\chi_{\left(1,62\right)}^{2}=$0.14, *p* = 0.76]. Likewise, there were no significant differences between participants in terms of FCR/P scores [*t*_(60)_ = −0.26, *p* = 0.79].

### Satisfaction With the Booklet

Almost 75% (37/49) of the respondents rated the booklet to be relevant to people with ovarian cancer and indicated it provided the needed information about FCR/P (as indicated by ratings > 80/100). Only 1 woman indicated that the booklet was not at all relevant. More than two thirds of women (32/49) rated the booklet as at least moderately helpful (ratings > 50/100) in managing their worries about cancer coming back or progressing. Of those, 14/49 reported that it was completely helpful, and only 3/49 thought it was not helpful at all. Importantly, 93% (41/44 women) of the participants would recommend the booklet to other women.

### FCR/P Results

Self-reported outcomes on the FoP-Q indicated that, on average, women with ovarian cancer fell within the clinical range (*M* = 35.58, *SD* = 8.52). Based on the cut-off score on the FoP-Q of 34, 56% (*n* = 35/62) of the participants reported clinically significant levels of FCR/P and the remainder (44%; *n* = 27/62) reported FCR/P scores within the normal range.

Overall, significant differences were not observed in the FoP-Q scores before (*M* = 35.4, *SD* = 8.59) compared to 1 week after reading the booklet (*M* = 33.94, *SD* = 9.00) [*t*_(49)_ = 1.71, *p* = 0.09; Cohen's *d* = 0.17; 95% CI −0.22 – 0.55], indicating that the booklet did not change levels of FCR/P. In considering whether the booklet had a differential impact based on level of FCR/P, we conducted a 2 × 2 mixed-model ANOVA. Consistent with the *t*-test reported above, there was no significant main effect of time [*F*_(1,48)_ = 2.69, *p* = 0.11] on FCR/P scores. There was a significant main effect of FCR/P level indicating that women scoring in the clinical range had higher levels of FCR/P throughout the study [*F*_(1,48)_ = 81.96, *p* > 0.001]. The interaction between time and FCR/P level indicated that clinical status did not impact the effect of time on FCR/P scores [*F*_(1,48)_ =0.13, *p* = 0.72].

Finally, we performed Pearson product-moment correlations to investigate the relationships between FCR/P and ratings of satisfaction. There was no significant correlation between ratings of satisfaction of the booklet in terms of providing sufficient information and level of FCR/P (*r* = −0.24, *p* = 0.10). However, correlations indicated that women with higher levels of FCR rated the booklet as less helpful in managing their worries about FCR/P (*r* = −0.316, *p* = 0.03).

## Discussion

The aim of this study was to determine whether an online booklet about FCR/P led to reductions in FCR/P and whether women were satisfied with the resource. The results demonstrated that there were high levels of satisfaction, and that most women would recommend the booklet to others. However, the booklet did not significantly improve levels of FCR/P, nor did it worsen them. The impact of the booklet on FCR did not differ for women in the clinical range for FCR/P compared to those with lower levels of FCR/P, although women with higher FCR/P rated the booklet as less helpful. Taken together, these results suggest that women believed that the booklet provided relevant information and was helpful, but the booklet was insufficient to reduce FCR/P.

These results are not entirely inconsistent with the previous literature and there are a number of potential reasons that might account for the failure to find an effect of this online resource. Firstly, Cuthbert et al. (2019) found mixed effects of self-help interventions, with some studies finding an effect and others not. They noted that very few self-help resources included specific behavior change techniques (e.g., Michie et al., 2011) and this could account for the failure of some interventions to affect change. This is true of the online resource in this study, which did not specifically include behavior change techniques.

Secondly, Cuthbert et al. (2019) described that in many self-help resources, there was an absence of a theoretical basis for the information provided. The information in the current booklet was adapted from the ConquerFear program (Butow et al., 2017), which was based on Fardell et al. (2016) model of the development of persistent FCR/P. This was the same model that was used as the first stage of the stepped care package developed by Lynch et al. (2020) for melanoma survivors who had responded to immunotherapy. However, in that study, the authors also included exercises as well as information, and there were three brief telephone conversations. Nevertheless, results on the FoP-SF-Q in the FearLESS study were similar to our results. Lynch et al. (2020) did not report the significance of their results for the 21 people that completed the self-help component, but the Cohen's *d* was similarly small (*d* = 0.02, 95% CI −0.59 – 0.62). Thus, even though both interventions were based on a theoretical model, neither appeared able to change FCR/P significantly and therefore this does not appear to explain the lack of effect observed here.

Thirdly, it has been suggested that some level of FCR/P is adaptive for people following cancer (Butow et al., 2018). This is because for all people who have been diagnosed with cancer, a recurrence is possible. For those in our study, with ovarian cancer, this is particularly the case since up to 70% of women with ovarian cancer will have a recurrence. According to this argument, FCR/P can provide the motivation to adhere to surveillance and therefore identify when a recurrence occurs. While this explanation cannot be excluded, it should be noted that in the Tauber et al. (2019) meta-analysis, there was no effect of cancer stage on the efficacy of interventions for FCR/P. Nevertheless, the bulk of the research on FCR/P involved patients whose cancer has been treated with curative intent and are currently disease-free. More research is needed to determine whether FCR/P is similar in patient groups with poorer prognosis to determine whether similar approaches are indicated. It may be in samples with advanced disease and high risk of relapse that distress and/or QOL are more relevant outcomes than FCR/P.

Finally, it is likely that the simple static FCR/P booklet, available in a PDF, was not sufficient to bring about change for the women who accessed it through this study who had high levels of FCR/P. FCR/P levels that were demonstrated by women in this study can be persistent and very distressing. It is perhaps unsurprising that a brief resource would not be sufficient to reduce FCR/P when one considers that even amongst the 8 available RCTs of psychological interventions with FCR as primary target, the effects were relatively small (Cohen's *d* = 0.44) (Tauber et al., 2019). However, it does pose a problem. With the increasing number of survivors, the small psycho-oncology workforce and the high levels of FCR/P, how can we meet the needs of survivors for help managing FCR?

We urgently need to focus on research that can develop cost-effective interventions that can be implemented in practice. Both the ConquerFear and SWORD studies (Butow et al., 2017; van de Wal et al., 2017) were shown to be cost effective, in that they had reasonable willingness to pay thresholds. However, we also need to consider stepped care models, such as FearLESS (Lynch et al., 2020), which have less time intensive interventions (such as self-management components that can be delivered via internet or telehealth) and/or utilize other members of the oncology workforce. Liu et al. (2019) in their review, concluded that there was insufficient evidence to support the delivery of interventions by non-specialists. However, there have been successful applications of nurse-led approaches, or clinician-driven interventions (Humphris and Ozakinci, 2008; Davidson et al., 2018; Reb et al., 2020). This needs to be a priority for research, particularly as patients themselves are more likely to take up the offer of therapy with nurses than with psychologists or psychiatrists (Brebach et al., 2016).

### Study Limitations

A number of methodological limitations are to be noted in the current study. Firstly, we did not recruit participants from clinical services and so relied on self-report regarding medical details. We did not take into account specific anxiety provoking situations such as oncology or scanning appointments. Studies have consistently shown that the time period when scan results are due can trigger significant anxiety in some patients (Feiler, 2011). This was not assessed and may have impacted the levels of FCR/P for some participants. Secondly, we are uncertain as to how much the booklet was read prior to the follow-up survey and the time was 1 week, and it might take longer for women to process apply the information, or it may have had immediate effects that tapered over time. The levels of motivation and engagement of the participants with the material could vary and could possibly provide a partial explanation for the results. Unfortunately we were unable to get data on how often women downloaded the booklet or how long they used it for. We did not have the pamphlet assessed formally by experts, which may have improved the resource and led to higher satisfaction. Further, our sample included all English-speaking participants and we were unable to get information about women that chose not to take part, therefore, the generalizability of this online resource across people from diverse backgrounds is unknown. The study would have benefitted from a formal power analysis since the study only had sufficient power to detect a moderate effect size (Cohen's *d* = 0.33). Finally, we developed a satisfaction scale for the study rather than using a previously validated scale.

### Implications

Findings of the present study suggest that we need to develop brief interventions that are scalable to try and help manage the demand for support for FCR. Stepped care models, such as the FEARLESS (Lynch et al., 2020) approach are likely to be important, but we need evidence to support the efficacy of the first step. Internet-delivered approaches would be an obvious first step, however, the first of these to be trialed produced null findings (van Helmondt et al., 2020), and the only other reported intervention, iConquerFear (Smith et al., 2020) is in the process of being evaluated (Lyhne et al., 2020). In the most recent meta-analysis of treatment for FCR (Tauber et al., 2019), only two minimal interventions were identified. One of these, gratitude training improved well-being and had an impact on some aspects of FCR (Otto et al., 2016). The other intervention trailed was cognitive bias modification (CBM). CBM has been found to be effective in anxiety (Jones and Sharpe, 2017) and has shown some promise in managing some aspects of FCR/P (Lichtenthal et al., 2017). To be able to meet the growing needs of survivors to help them manage FCR/P, there is an urgent need to develop minimal interventions that are efficacious. If effective minimal interventions can be developed, they could be a useful addition to a stepped care approach in reducing FCR/P.

## Conclusion

In conclusion, the online resource developed for women with ovarian cancer was rated as helpful. Women reported high levels of satisfaction and almost all women reported that they would recommend the resource to a friend. Despite these positive findings, the online resource did not lead to reductions in FCR/P and importantly it was those women with the highest levels of FCR/P who found the resource least helpful. Future research needs to investigate ways in which interventions can be delivered to the large number of cancer survivors who need help to deal with FCR/P.

## Data Availability Statement

The raw data supporting the conclusions of this article will be made available by the authors, without undue reservation.

## Ethics Statement

The studies involving human participants were reviewed and approved by University of Sydney's Human Research Ethics Committee (Project No: 2018/993). The patients/participants provided their written informed consent to participate in this study.

## Author Contributions

PP, LS, and HR conceived the idea for the present study. PP wrote the first draft of the manuscript and performed the data analysis. PB and AS contributed in developing the online booklet. HR contributed to the participant recruitment. All authors discussed the results and contributed to the final manuscript.

## Conflict of Interest

The authors declare that the research was conducted in the absence of any commercial or financial relationships that could be construed as a potential conflict of interest.

## Footnotes

^1^Measures of interpretation bias and physical symptoms were included, the results of which are presented elsewhere.
